# Hyaluronan in COVID-19: a matrix for understanding lung disease

**DOI:** 10.1128/mbio.02609-24

**Published:** 2024-11-18

**Authors:** Rebecca J. Dodd, Judith E. Allen, Anthony J. Day

**Affiliations:** 1 Wellcome Centre for Cell-Matrix Research, Faculty of Biology, Medicine and Health, University of Manchester, Academic Health Science Centre, Manchester, United Kingdom; 2 Lydia Becker Institute of Immunology and Inflammation, University of Manchester, Academic Health Science Centre, Manchester, United Kingdom; Johns Hopkins University, Baltimore, Maryland, USA

**Keywords:** lung infection, extracellular matrix, hyaluronan

## Abstract

The polysaccharide hyaluronan (HA) is an important component of lung extracellular matrix that increases following infection with influenza or severe acute respiratory syndrome coronavirus 2 (SARS-CoV-2). Hellman et al. (U. Hellman, E. Rosendal, J. Lehrstrand, J. Henriksson, et al., mBio 15:e01303-24, https://doi.org/10.1128/mbio.01303-24) show that fragmented HA accumulates in the lungs of coronavirus disease 2019 (COVID-19) patients, with systemic levels of HA being associated with reduced lung function 3–6 months after infection. This study provides novel insights into HA’s role in COVID-19 pathology and its potential utility as a biomarker for disease severity. However, much remains to be understood about the lung HA matrix in COVID-19 and how it compares to other lung conditions. In particular, the role of HA-binding proteins in organizing HA into a crosslinked network is yet to be fully determined at a molecular level. This knowledge is crucial in understanding the inter-relationships between the structure of the HA matrix and the regulation of the immune response, and thus our ability to target HA therapeutically for improved outcomes in COVID-19.

## COMMENTARY

Hyaluronan (HA) is a polysaccharide of very high molecular weight (HMW) found ubiquitously in the tissues of mammals. It is associated with the cell surface and acts as an essential component of the extracellular matrix. HA synthesis is induced in the lung in response to injury (1), and its levels increase in asthma and on infection with influenza, respiratory syncytial virus, and severe acute respiratory syndrome coronavirus 2 (SARS-CoV-2) (2–8).

In the healthy lung, HA is present in the airway basement membrane, glycocalyces of the alveolar and bronchial epithelium, and blood vessel endothelial cells (1, 9). However, upon injury, HA rapidly accumulates throughout the alveolar spaces of the lungs. Early reports during the SARS-CoV-2 pandemic, in particular from Urban Hellman and colleagues, highlighted increased HA within the lungs of deceased patients (6, 10). Following this, it was found that HA levels were greatly increased in the circulation of COVID-19 patients, as well as in the lungs, where in both cases this correlated with disease severity (7, 11). Studies in SARS-CoV2-infected mice revealed that the type 2 cytokine IL-13 has a critical role in driving HA accumulation (e.g., in the alveolar epithelium) (7).

Hellman et al. (12) built on their previous findings to show (using light-sheet microscopy) that in deceased COVID-19 patients (*n* = 3) there was a reduction in the alveolar surface area compared with lung resections from healthy controls (*n* = 4) . They found that HA was present in thickened alveolar walls and within alveolar secretions, whereas collagen staining was associated with irreversibly damaged alveoli. Both of these tissue changes likely play a key role in the reduced lung function that has been observed long-term in COVID-19 patients (13, 14). Hellman et al. (12) also extracted HA from the lungs and found that it was highly fragmented in all of the samples from deceased COVID-19 patients (*n* = 5; necropsy tissue), with a large proportion below 30 kDa (compared to >4,500 kDa in biopsies from healthy controls [*n* = 5]). However, in nasopharyngeal and endotracheal aspirates from COVID-19 patients (*n* = 3), with severe disease, both the amount of HA and the extent of fragmentation were much more variable. Interestingly, in a model of influenza in mice, there was no significant change in the size distribution of extracted lung HA at the peak of infection compared with uninfected controls (15). Together, these data suggest that temporal changes to HA size may be an important aspect of the tissue response to infection, and further work is clearly required to understand HA metabolism over the time course of COVID-19 and other lung pathologies. In this regard, HA fragments in the circulation have been associated with organ failure in COVID-19 patients (11), illustrating the importance of this question.

As highlighted by Hellman et al., low-molecular-weight (LMW) HA is often considered to be pro-inflammatory. Indeed, their study shows an increased influx of neutrophils in COVID-19 lungs (12), although whether this is directly related to the fragmentation of HA is unclear. Previous studies have suggested that LMW HA increases inflammation through TLR-mediated pathways (reviewed in eference 16). However, some of the underpinning experimental data are confounded by the presence of lipopolysaccharide in the HA preparations that were used (17). Furthermore, there are other potential explanations as to how LMW HA may contribute to pro-inflammatory signaling (16). For example, LMW has been shown to be a highly effective competitor of HMW HA binding to the major HA receptor, CD44 (18), i.e., removing possible anti-inflammatory effects of HMW HA (19).

**FIG 1:**
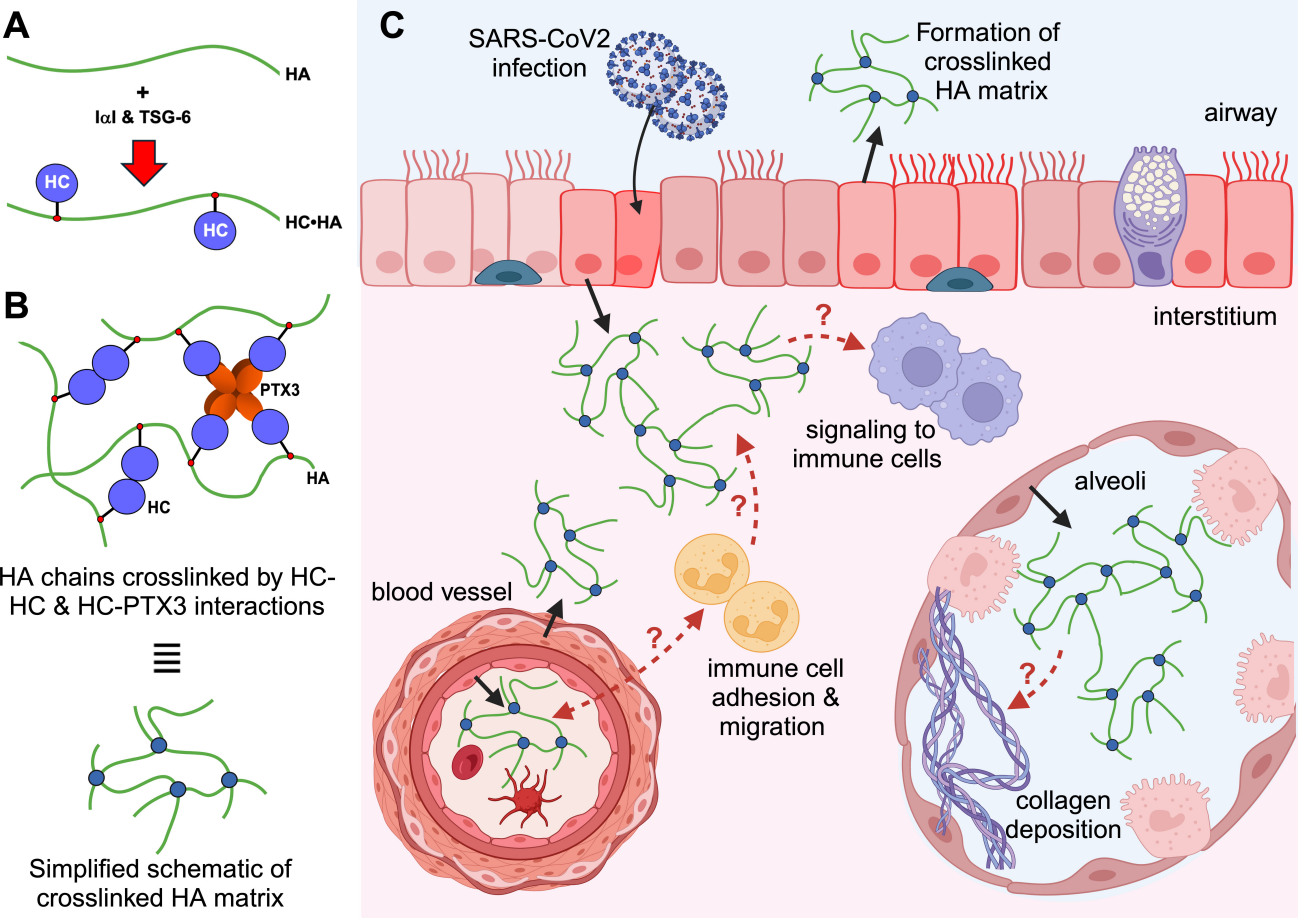
Putative roles of HC•HA matrices in the SARS-CoV-2-infected lung. (**A**) HA becomes covalently modified, in a TSG-6-dependent manner, with HCs from IαI proteoglycans to form HC•HA complexes during inflammation (see reference 23)). (**B**) Through a series of non-covalent HC-HC and HC-PTX3 interactions, HC•HA and accessory proteins such as PTX3 are incorporated into a crosslinked HA matrix (16). (**C**) We hypothesize that HC•HA-containing matrices will be found throughout the lung following SARS-CoV-2 infection, including in the airways, alveolar spaces, and interstitium. The exact composition and function, for example, in the different locations, remain to be determined. Figure created using BioRender.com.

Hellman et al. (12) demonstrated that HA was extensively deposited throughout the alveolar structures of infected lungs, consistent with previous studies (8). However, the composition of the HA matrix in the lung post-SARS-CoV-2 infection remains poorly characterized. In the extracellular matrix of most tissues, HA is associated with HA-binding proteins that dictate the organization of the HA biopolymer and thus the overall structural and functional properties of the HA matrix formed (16). For example, the covalent modification of HA with “heavy chains” (HCs) from the inter-alpha-inhibitor (IαI) family of proteoglycans (Fig. 1A) is an understudied hallmark of tissue inflammation (3, 20–22), where the HC•HA complexes formed (Fig. 1B) can be pro- or anti-inflammatory depending on composition and context (16, 23). HC•HA is detectable in plasma from SARS-CoV-2-infected patients (11), and our own studies indicate that HA modified with HCs is present in the lung tissue from SARS-CoV-2-infected mice (R. J. Dodd, D. Hart, J. E. Allen, A. J. Day, W. A Petri, and T. E. Sutherland, unpublished data). However, the exact composition of these matrices remains to be determined in COVID-19. Importantly, the HC•HA composition (Fig. 1C) likely affects the adhesive and signaling properties of the HA matrix, particularly for immune cells (15, 16, 22). Four members of the HC family (HC1, HC2, HC3, and HC5) become attached to HA (23), and other constituents, such as pentraxin-3 (24) and versican (8), can play a major role in the crosslinking and expansion of the HA network (16). Pentraxin-3, for example, is expressed by lung macrophages and endothelial cells from COVID-19 patients, and its level in plasma acts as a strong prognostic marker for 28-day mortality (25). Therefore, temporal and spatial analyses of HA and its associated proteins are needed if we are to fully appreciate the role these matrices play in lung inflammation, resolution, and repair.

Hellman et al. (12) showed that HA was increased systemically in COVID-19 patients and circulating levels correlated with the severity of infection (12) as has been reported previously (7). Importantly, in their cohort, Hellman et al*.* showed that recovered patients had reduced (but not baseline) plasma HA during the convalescent phase and that plasma HA levels negatively correlated with lung diffusion capacity up to 6 months after infection. In a mouse model of influenza, HA was also elevated in the lungs at the 6-month timepoint (21), indicating that the long-term dysregulation of HA synthesis and turnover may be a common feature of virally induced lung disease. HA accumulation in the lung post SARS-CoV-2 infection in mice was shown to be IL-13-dependent and IL-13 neutralization reduced disease severity (7). A small randomized, placebo-controlled trial using dupilumab (an IL-4/IL-13-blocking antibody) in COVID-19 patients requiring hospitalization demonstrated reduced mortality (13), with follow-up studies showing that patients who received dupilumab had significantly improved lung function scores 1 year post treatment (14). Taken together, these studies indicate that reducing HA accumulation is likely to be beneficial in reducing the long-term complications of COVID-19.

In addition, to preventing HA biosynthesis via IL-13 blockade (13) (which warrants assessment in a larger clinical trial), there are alternative therapeutic approaches that have been suggested (see reference 26). These include the use of 4-methylumbelliferone to inhibit HA production in the lung (27), hyaluronidase to break down the pathogenic HA matrix (8, 21, 28), and even inhaled HMW HA (29) to harness HA’s anti-inflammatory effects. In all these approaches, the precise timing of when treatment is given is likely to be important in its effectiveness and safety. Additionally, a particular intervention may not be suitable for all COVID-19 patients, and this may be dependent on differential compositions of the lung HA matrix. Thus, while HA is clearly an attractive biomarker for COVID-19 severity, as demonstrated in the new paper from Hellman and colleagues (12), other components of the HA matrix may provide important additional information underpinning a personalized medicine approach in SARS-CoV2 infection as well as in other lung pathologies.
